# Pharmacological and clinical evaluation of deferasirox formulations for treatment tailoring

**DOI:** 10.1038/s41598-021-91983-w

**Published:** 2021-06-15

**Authors:** Andrea Piolatto, Paola Berchialla, Sarah Allegra, Silvia De Francia, Giovanni Battista Ferrero, Antonio Piga, Filomena Longo

**Affiliations:** 1 Department of Clinical and Biological Sciences, University of Torino, Torino, Italy; 2 Clinical Pharmacology, Department of Clinical and Biological Sciences, University of Torino, Turin, Italy

**Keywords:** Anaemia, Medical research, Drug safety, Clinical pharmacology, Pharmacodynamics, Pharmacokinetics

## Abstract

Deferasirox (DFX) is the newest among three different chelators available to treat iron overload in iron-loading anaemias, firstly released as Dispersible Tablets (DT) and more recently replaced by Film-Coated Tablets (FCT). In this retrospective observational study, pharmacokinetics, pharmacodynamics, and safety features of DFX treatment were analyzed in 74 patients that took both formulations subsequently under clinical practice conditions. Bioavailability of DFX FCT compared to DT resulted higher than expected [C_max_: 99.5 (FCT) and 69.7 (DT) μMol/L; AUC: 1278 (FCT) and 846 (DT), P < 0.0001]. DFX FCT was also superior in scalability among doses. After one year of treatment for each formulation, no differences were observed between the treatments in the overall iron overload levels; however, DFX FCT but not DT showed a significant dose–response correlation [Spearman r (dose-serum ferritin variation): − 0.54, P < 0.0001]. Despite being administered at different dosages, the long-term safety profile was not different between formulations: a significant increase in renal impairment risk was observed for both treatments and it was reversible under strict monitoring (P < 0.002). Altogether, these data constitute a comprehensive comparison of DFX formulations in thalassaemia and other iron-loading anaemias, confirming the effectiveness and safety characteristics of DFX and its applicability for treatment tailoring.

## Introduction

Iron chelation therapy (ICT) represents a cornerstone in the management of iron overload in patients affected by thalassaemia and other iron-loading conditions, with a significant impact on morbidity and mortality^1,2^. Among the available drugs, deferasirox (DFX) represents the first-line iron chelator worldwide for the treatment of chronic iron overload due to blood transfusions in patients from the age of 2 years and older and for non-transfusion-dependent thalassaemia syndromes in patients aged 10 years and older^3,4^.

DFX is a tridentate ligand, with high affinity and specificity for iron. The active form is highly lipophilic and bound almost exclusively to serum albumin^5,6^. After absorption, the time to reach maximum plasma concentration (T_max_) is 1–4 h postdose; maximum plasma concentration (C_max_) and Area Under the concentration Curve over 24 h (AUC) linearly increase with dosage after a single administration and under steady-state condition. Its long half-life (t_1/2_, 11–19 h) allows a once-daily oral regimen. The main pathway of DFX metabolism is via glucuronidation by uridine diphosphate glucuronosyltransferase (UGT); a residual 6% of the prodrug is metabolized by cytochrome P450 (CYP) 1A1 and 2D6^6^. The majority (84%) of DFX and its metabolites is eliminated via bile through multidrug resistance protein 2 (MRP2) and 8% by breast cancer resistance protein (BCRP)^6,7^; the remaining 8% is excreted in the urine. DFX is known to interact with other drugs (e.g. rifampicin), that could alter significantly its bioavailability^8^.

Extensive clinical trial programs in adult and paediatric patients with thalassaemia, myelodysplastic syndromes (MDS), sickle-cell disease, and other rare anaemias gathered data about the efficacy and the safety of DFX^3,9–11^, proving it to be comparable to the other chelators^3,10,12,13^ and demonstrating its undisputed advantage for patient compliance and adherence to long-term treatment^10,12–14^. However, increasing evidence suggests that some patients under DFX treatment do not achieve adequate chelation, even if receiving a maximal dose of the drug (poor responders). A small-scale study observed that poor responders had significantly lower systemic drug exposure compared to controls, identifying the difference in bioavailability to be the main cause of this effect^15^. Further studies identified polymorphism in UGT1A1, CYP1A1 and CYP1A2 to be predictive factors of inadequate response to DFX treatment^16^.

DFX long-term administration is known to be associated with various adverse events and mild to severe alterations of hepatic and kidney functions are the most relevant among them. Reports of drug-induced liver injury, including hepatic failure, have been reported during post-approval use of this compound, showing a hepatocellular pattern of injury with marked elevations in serum aminotransferase in the most severe cases^17,18^. A reversible, mild increase in serum creatinine (sCr) has been reported since the core registration trials^19^; successively, acute nephrotoxicity related to DFX was reported in cases of proximal tubulopathy and Fanconi syndrome or acute interstitial nephritis. Due to the risk of serious adverse reactions, including acute kidney injury (AKI), hepatic failure, and gastrointestinal (GI) haemorrhage, strict monitoring of renal and hepatic function is recommended when administering DFX^4^. A comprehensive analysis requested by the FDA Paediatric Advisory Committee established a clear relationship between high-dose DFX [> 30 mg/kg/day (DT) or > 21 mg/kg/day (FCT or sprinkle granules)], low ferritin (< 1000 μg/L) and the risk of AKI. Analyses of pooled data from three clinical studies found that small decreases in estimated glomerular filtration rate (eGFR) among paediatric patients due to small body surface area are associated with significantly increased DFX plasma concentrations, possibly leading to severe nephrotoxicity in case of over-chelation^20^.

To overcome the GI tolerability and palatability issues of the first licensed preparation in Dispersible Tablets (DFX DT), a Film-Coated Tablets formulation (DFX FCT) was released in 2016 and is now available in most countries. The new formulation allows a single-step administration (without preparation through water dispersion) and less stringent food restrictions compared to DFX DT^4^. Due to higher bioavailability, the new formulation contains 30% less active principle. The lack of food effect on the bioavailability of DFX FCT formulation is expected to improve adherence to the therapy schedule and lead to more predictable drug exposure. The randomized, open-label, phase II ECLIPSE study compared original DFX DT formulation with the dose-adjusted FCT in patients with thalassaemia and lower-risk MDS and demonstrated similar safety profiles with fewer tolerability issues, together with greater satisfaction and palatability in the FCT group. The imbalance in reported renal events observed between the two treatment arms was likely attributed to a larger proportion of patients receiving a higher than recommended dosage in the FCT group, as well as non-adherence to protocol-recommended dose modifications^21^. Since the recent introduction of the new formulation, only a few data were reported about long-term effects of DFX FCT outside clinical trial settings^22–24^, and very few independent comparisons of the two formulations are currently available.

## Design and methods

### Ethics declarations

The study was approved by the local ethical committee (*Comitato Etico Interaziendale A.O.U. San Luigi Gonzaga di Orbassano e AA.SS.LL. TO3-TO4-TO5*, Registration Number 79/2012) and performed in accordance with Good Clinical Practice guidelines and the Declaration of Helsinki. DFX PK curve assessment has been approved by the hospital licensing committee and included in the clinical routine, fully reimbursed by the public health system. Each patient or legal guardian signed the informed consent.

### Study design

A retrospective observational study was designed based on clinical records of procedures performed for clinical practice and available at Haemoglobinopathies Centre in Orbassano (To), Italy, from 01 January 2011 to 31 March 2019. The aim of the study was to compare pharmacokinetics (PK), pharmacodynamics (PD) and safety of both DT and FCT DFX formulations in a group of patients with thalassaemia and other congenital anaemias. Two separate and subsequent sections were considered and compared to each other: one accounting for data about DFX DT and the other accounting for data about DFX FCT (Suppl. Fig. 1). Each patient took part in both of these sections, so that measures obtained for DT were compared with those obtained for FCT for every participant. All patients were evaluated for PK; in addition, a subgroup defined by inclusion and exclusion criteria was evaluated for PD and safety. The study time was not selected previously and was therefore determined by the available clinical records in the time frame considered.

### Inclusion and exclusion criteria

**Figure 1:**
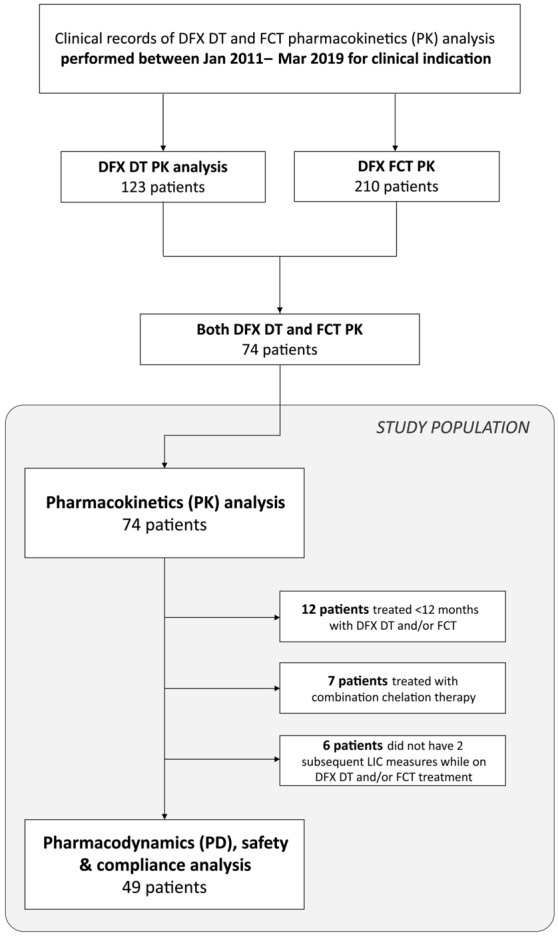
Study Population. Deferasirox (DFX) pharmacokinetic (PK) tests performed for clinical practice between 01 January 2011 and 31 March 2019 were considered. 74 patients who performed one PK tests for DFX dispersible tablets (DT) and one for film-coated tablets (FCT) formulation formed the study population for the PK analysis. Among them, only those who completed at least 1 year of treatment with each formulation alone were further included for the pharmacodynamics and safety analysis of this study.

To be included in this study, patients must have had a diagnosis of hereditary iron-loading anaemia or other red blood cell disorders requiring iron chelation therapy. In addition, they must have had a record of at least one PK evaluation for both DFX DT and FCT in the period considered for the study. Patients that performed ≥ 12 months monotherapy with DFX DT and ≥ 12 months monotherapy with DFX FCT and performed ≥ 2 Liver Iron Concentration (LIC) measurements for each treatment period (± 3 months from the time of drug administration, performed ≥ 6 months apart) were included in the analysis of PD, safety and compliance. Patients treated by administration of any other iron chelator than DFX at the time of evaluation were included only in the PK analysis and excluded from the study of PD, safety and compliance. (Fig. 1).

### Patient demographics and clinical characteristics

Data about personal and clinical condition (gender, age, Body Mass Index (BMI), state of pubertal activation according to Tanner’s scale), were collected for all patients from the clinical records available in the study period. Diagnoses were collected from clinical records. Diagnoses of β-thalassaemia were classified as thalassaemia major or intermedia, according to their clinical presentation (β-thalassaemia major entails lifelong regular transfusion requirement while thalassaemia intermedia is referred to patients who do not require transfusions or may require occasional or even frequent transfusions for defined periods of time). DFX DT (Exjade, Novartis Pharma) and DFX FCT (Exjade, Novartis Pharma) were administered as standard of care in successive and separate times. Data about administered doses were collected from clinical records.

### Pharmacokinetics

PK study was performed in a controlled setting, where patients underwent ≥ 48 h washout from last dose of ICT, if not naive. Possible confounding or PK-altering factors, such as occasional administration of non-steroidal anti-inflammatory drugs or ethanol consumption were prohibited for ≥ 48 h before performing PK. A trained nurse supplied patients with the indicated dose of DT formulation, carefully ensuring a proper dispersion and administration, while FCT was supplied directly in the required amount of tablets. Patients were fasting before DT administration, while a light meal was allowed according to each patient’s preference and usual custom for FCT. Serum DFX concentrations were measured by HPLC–UV method, as previously described^25^. Briefly, a simple protein precipitation extraction procedure was applied on 500 µL of plasma aliquots: 100 µL of internal standard (IS) working solution, made at the final concentration of 100 µg/mL in methanol and used immediately, was added to plasma sample. Then 750 µL of protein precipitation solution (methanol:acetonitrile 50:50, v/v) was added to each sample. After brief mixing (30 s), samples were centrifuged at 12,000 rpm for 15 min and 800 µL of the obtained supernatant were transferred to vials, for injection in column (20 µL). Chromatographic separation was achieved on a C18 reverse phase column and eluate was monitored at 295 nm, with 8 min of analytical run. Quantification of DFX was performed by IS use: calibration curves, over the concentration range chosen, were built with the spike height ratios of each standard calibration and IS, and fitted using linear regression. This method has been validated following Food and Drug Administration procedures: mean intra and inter day variability was 4.64 and 10.55%; mean accuracy was 6.27%; mean extraction recovery 91.66%. Calibration curves ranged from 0.078125 to 40 µg/mL. Limit of quantification was set at 0.15625 while limit of detection at 0.078125 µg/mL. For each patient, a 5-timepoints PK curve was obtained, measuring DFX plasma concentration at 0 h (before administration) and 2 h, 4 h, 6 h and 24 h after administration. Maximum plasma concentration (C_max_) and Area Under Curve (AUC) were calculated accordingly.

### Pharmacodynamics and safety

Serum ferritin (FTN) determined by chemiluminescence and Liver Iron Concentration (LIC) measured by superconducting quantum interference device (SQUID) were collected from the clinical records available in the study period. The single reported value of FTN is calculated as the mean of all FTN values available in the time ± 1.5 months from the date of LIC measure. In order to account for different times of observation and different conditions at baseline for each patient, the rate of variation of FTN and LIC (ΔFTN and ΔLIC, respectively) was considered instead of the absolute difference. Briefly, the difference between baseline and end-of-study values of FTN and LIC was normalized for the actual days of observation). For patients transfused during the study period, mean iron intake of the previous year was calculated from their clinical records, based on the number of RBC units received, using the formula: *transfusion volume x haematocrit* *x 1.08*, as reported previously^26,27^. Compliance was assessed from patients’ self-report and validated by drug accountability and physicians’ estimation. Concomitant medications administered in the study period were collected from clinical records. Data regarding safety (alanine aminotransferase (ALT), aspartate aminotransferase (AST), sCr, protein/creatinine ratio) were collected from all clinical records available in the study period. eGFR was calculated using Cockcroft-Gault and Bedside Schwartz formula for patients aged ≥ 14 and < 14 years, respectively. Acute Kidney Injury (AKI) was defined according to guidelines as previously described^20^, specifically, an episode of AKI was reported if reaching any of the following thresholds: eGFR < 90 mL/min/1.73 m^2^ if baseline ≥ 100 mL/min/1.73 m^2^ or eGFR < 75% of baseline if otherwise and any case of eGFR < 60 mL/min/1.73 m^2^). Chronic Kidney Disease (CKD) risk was defined according to guidelines^28^. For PD and safety analysis, study time was defined as the period between two subsequent LIC measures, considered as the treatment period. The end of study was defined as the last measure obtained within the treatment period.

### Statistical analysis

Continuous variables were described by mean and standard deviation and categorical variables by frequency and percentage. Wilcoxon matched-pairs test and Spearman's r index were used to analyze differences and correlations between DFX DT and DFX FCT treatments. Exact Clopper–Pearson test was used to analyze differences between probabilities of events. A significance threshold of 0.05 was considered for all tests. A quantile regression was used to analyze the correlation between PK or PD parameters and dose adjusted by age, BMI, sex and pubertal activation. Median regression was used in this analysis. The quantile regression was chosen to avoid data transformation due to the non-normal distribution of the errors and 95% confidence intervals were estimated by bootstrapping (500 samples). Statistics were calculated using Statsoft Statistica 10 or R software version 4.0.2^29^.

## Results

### Patients' characteristics

In the period considered, 123 and 210 patients underwent at least one PK evaluation for DFX DT and FCT administration, respectively. Seventy-four patients diagnosed with β-thalassaemia major (61), β-thalassaemia intermedia (11), Diamond-Blackfan anaemia (1) and Stomatocytosis (1) met inclusion criteria for PK analysis; among them, 36 (49%) were female. Mean age was 32 ± 11 years at DT and 37 ± 11 years at FCT study time (Table 1). Nine were paediatrics. Forty-nine out of 74 patients were subsequently included in PD and safety analysis. Twenty-five were excluded according to exclusion criteria (Fig. 1). Mean observational study time was 11.6 ± 3.5 and 11.3 ± 3.3 months per patient for DT and FCT, respectively, corresponding to a total time of 47.5 (DT) and 46.1 years (FCT). Actual administered dose was adjusted during the study time when clinically indicated; however, no statistically significant differences were observed between doses tested at PK and mean administered dose. No patients had a prescription for any drug known to potentially modify DFX plasma concentration.

**Table 1:** Patient's characteristics.

	N	DT		FCT	
		Mean	SD	Mean	SD
**Pharmacokinetic (all patients)**					
Sex (F:M)	36:38	–	–	–	–
Age (years)	74	32.3	11.4	36.9	11.3
BMI (kg/m^2^)	74	20.9	3.8	21.7	4.6
**Pharmacodynamics and safety subgroup**					
Sex (F:M)	23:26	–	–	–	–
Age (years)	49	30.3	11.5	35.5	11.6
BMI (kg/m^2^)	49	21.0	3.5	21.6	3.8
Pubertal activation (Y:N)	49	–	–	–	–
Baseline FTN (ng/mL)	44 (DT) 49 (FCT)	1360	1246	1391	1504
Baseline LIC (mg Fe/g dw)	49	7.2	5.9	7.9	5.2
Baseline ALT (U/L)	49	55	76	26	25
Baseline AST (U/L)	49	40	39	31	13
Baseline sCr (mg/dL)	49	0.69	0.16	0.9	1.3
Baseline P/C ratio	48 (DT) 49 (FCT)	0.18	0.10	0.17	0.12

Basic and clinical patient’s characteristics registered at baseline for both treatments are showed in the table.

*DT* dispersible tablets, *FCT* film coated tablets, *FTN* serum ferritin, *LIC* liver iron concentration, *ALT* alanine aminotransferase, *AST* aspartate aminotransferase, *sCr* serum creatine, *P/C ratio* urinary protein to creatinine ratio.

### Pharmacokinetics analysis

**Figure 2:**
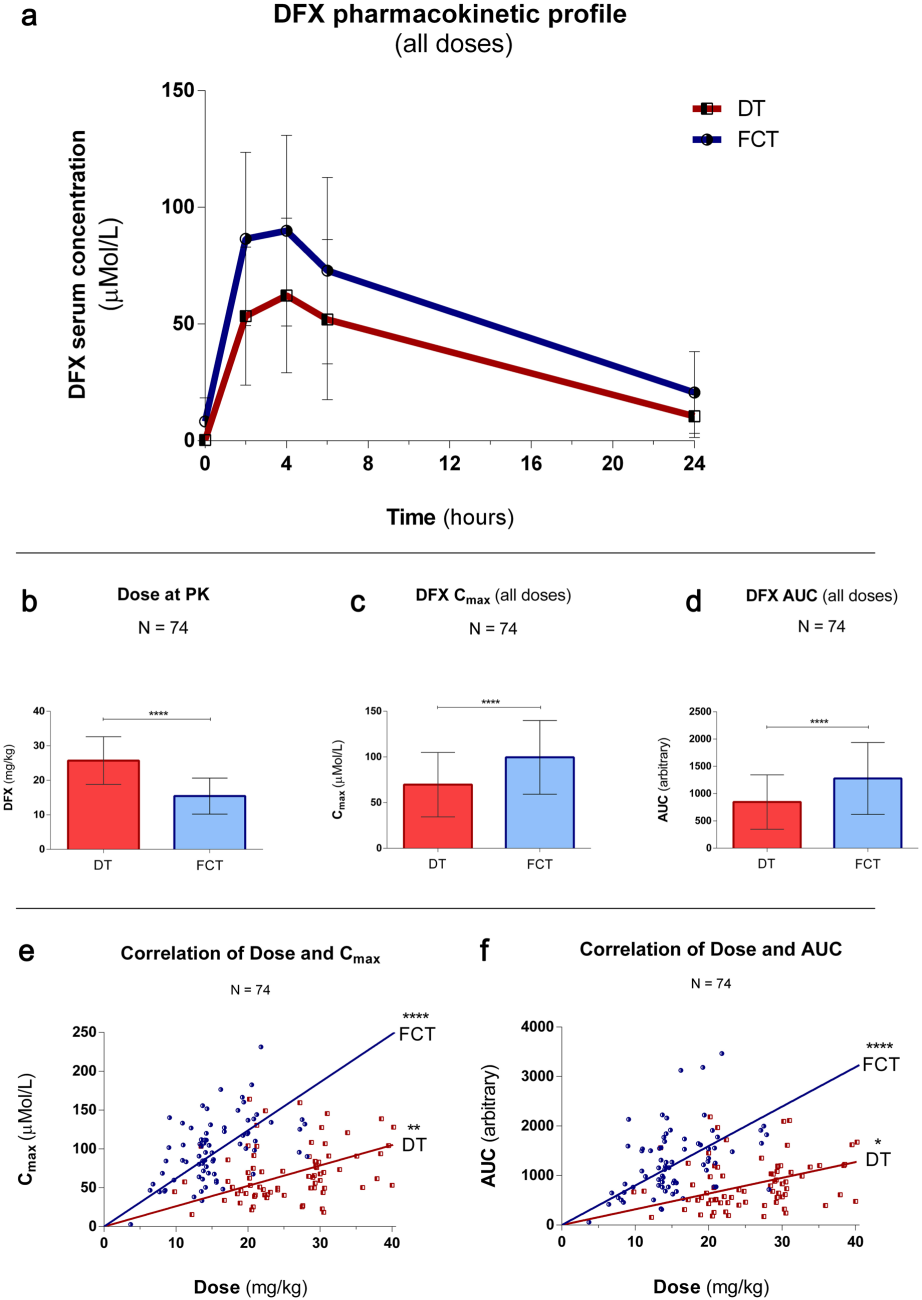
Pharmacokinetics: (**a**) Mean PK curve at all doses, for DFX DT and FCT. Blood samples were obtained pre-dose and at 2, 4, 6 and 24 h post dose administration. Mean and standard deviation are shown. (**b–d**) DFX was tested at a mean dose of 26.1 ± 6.9 (DT) and 15.5 ± 5.2 (FCT) mg/kg (− 41%), resulting in a maximum plasma concentration (C_max_) of 71.0 ± 35.2 (DT) and 101.5 ± 40.3 (FCT) µMol/L (+ 43%) and an Area Under the Curve (AUC) of 855.0 ± 499 (DT) and 1301.3 ± 658 (+ 52%). Error bars: standard deviation. (**e**) C_max_ correlated significantly with dose for both formulations, but the observed correlation was stronger for FCT: Spearman's r (dose-C_max_) = 0.32 (P = 0.006) and 0.53 (P < 0.0001) for DT and FCT, respectively. (**f**) AUC correlated significantly with dose for both formulations, but the observed correlation was stronger for FCT: Spearman's r (dose-AUC) = 0.27 (P = 0.02) and 0.45 (P < 0.0001) for DT and FCT, respectively.

When performing PK measurement, DFX testing dose was established for each patient according to their clinical condition. Overall, it was tested at a mean dose of 26.1 ± 6.9 and 15.5 ± 5.2 mg/kg for DT and FCT, respectively. FCT dose tested at PK was therefore 41% lower than DT. Despite the lower dose tested, both C_max_ and AUC were significantly superior in the FCT formulation, and specifically mean C_max_ measured 71.0 ± 35.2 (DT) and 101.5 ± 40.3 (FCT) μMol/L (P < 0.001) and AUC measured 855.0 ± 499 (DT) and 1301.3 ± 658 (FCT) (P < 0.001). (Fig. 2A–D). Variation coefficients of the mean measured 50.6% (DT) and 40.5% (FCT) for C_max_ and 58.9% (DT) and 51.5% (FCT) for AUC. Similar results were obtained when considering patients with different diagnoses in separate groups, i.e. thalassaemia major, thalassaemia intermedia and others. (Suppl. Fig. 2 + Suppl. Tab. 1). Successively, the correlation between the dose administered at PK and bioavailability indexes was analyzed. Spearman’s r (dose-C_max_) measured 0.32 (P = 0.006) and 0.53 (P < 0.0001) and Spearman’s r (dose-AUC) measured 0.27 (P = 0.02) and 0.45 (P < 0.0001) for DT and FCT, respectively. (Fig. 2E,F). When considering each diagnosis separately, the highest correlation was observed for patients affected by thalassaemia intermedia, resulting in a Spearman’s r (dose-C_max_) of 0.93 (P = 0.0001). (Suppl. Tab. 2). To better evaluate factors that could act as modifiers of drug exposure, correlation with gender, age, state of pubertal activation, and BMI were also investigated. None of these factors contributed to a significant difference in DFX bioavailability (Suppl. Tab. 3).

### Pharmacodynamics analysis

**Figure 3:**
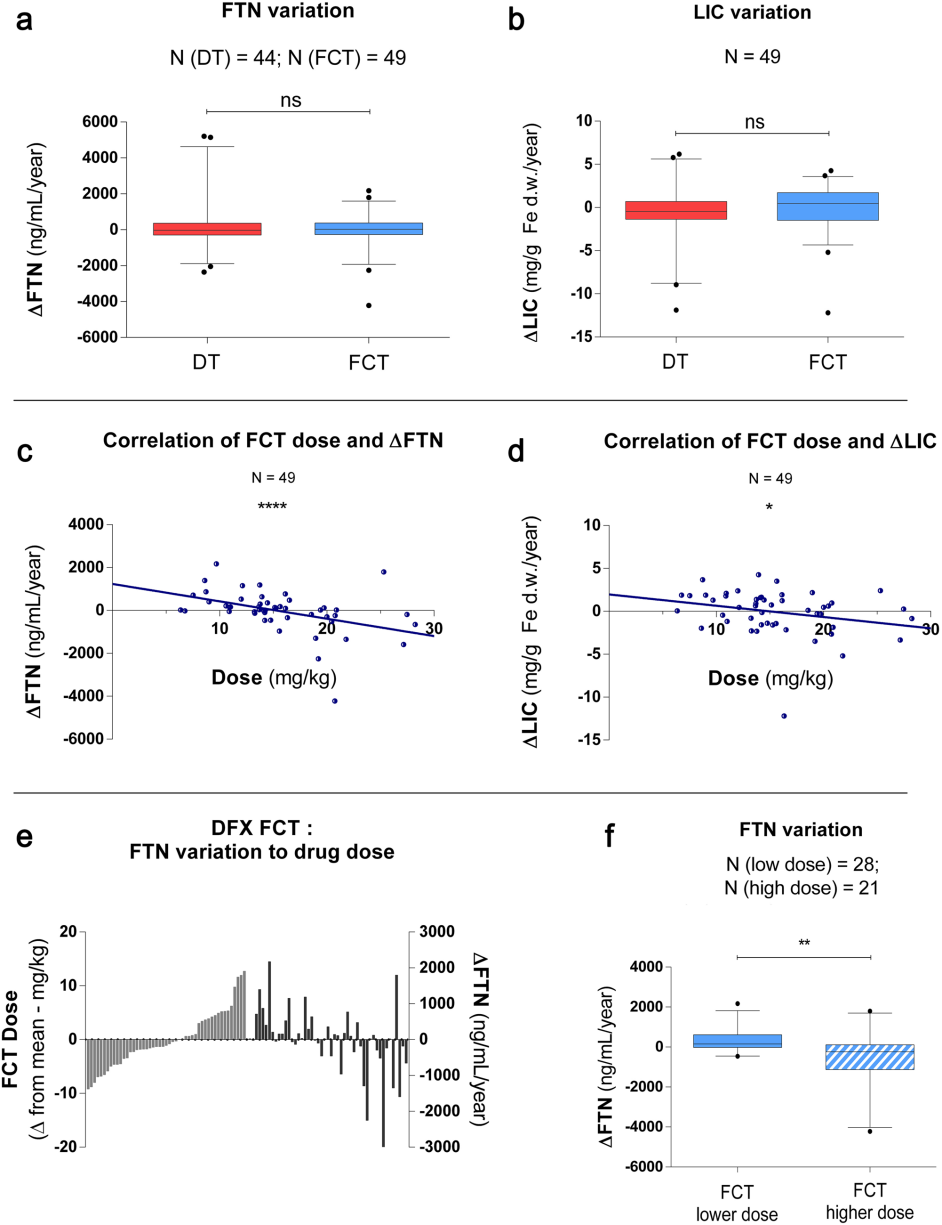
Pharmacodynamics. Iron overload changing during treatment measured by yearly variation of serum ferritin (ΔFTN) (**a**) and LIC (ΔLIC) (**b**) . Mean ΔFTN measured + 277 ± 1473 (DT) and − 24 ± 992 (FCT) ng/mL/year (*ns*); ΔLIC measured − 0.4 ± 3.2 (DT) and − 0.1 ± 2.7 (FCT) mg/g Fe d.w./year (*ns*). Correlation between administered dose of DFX FCT and iron overload variation measured both as (**c**) ΔFTN and (**d**) ΔLIC. Spearman's r (dose-ΔFTN) = − 0.54 (P < 0.0001) and Spearman's r (dose-ΔLIC) = − 0.32 (P = 0.02). (**e**) DFX FCT dose plotted as variation from the mean and correspondent ΔFTN variation for each patient. (**f**) ΔFTN in patients who received a DFX FCT dose lower (range 6.4–15.3 mg/kg/day; N = 28) or higher (range 15.6–28.2 mg/kg/day; N = 21) than the mean of all FCT doses considered in the study. (P = 0.001). Whisker plots show median (line), 25 to 75 percentile (box), 5–95 percentile (error bar) and individual results out of previous ranges (dots). *ns:* not significant.

To evaluate the effect of DFX administration over the treatment period, ΔFTN and ΔLIC levels registered for DT and FCT treatment were measured in a subset of patients. Baseline levels of iron overload were similar among treatment periods with each formulation. Specifically, FTN levels were not different, while LIC levels were slightly more elevated at the beginning of FCT study time (Table 1). No significant variations were observed for the whole group, nor for any of the diagnoses considered separately. (Fig. 3A,B, Suppl. Fig. 3 + Suppl. Tab. 4). The iron input calculated for those patients receiving blood transfusions did not differ significantly. (Suppl. Fig. 4).

The correlation among dose and ΔFTN or ΔLIC levels variation was assessed for both formulations, resulting in a significant dependence for FCT, but not for DT. Spearman's r (dose-ΔFTN) measured − 0.54 (P < 0.0001) and Spearman's r (dose-ΔLIC) measured − 0.32 (P = 0.02) for FCT formulation, while it was not significant for DT (Fig. 3C–E). This correlation was higher for patients affected by thalassaemia major [Spearman's r (dose-ΔFTN) measured − 0.69 (P < 0.0001) and Spearman's r(dose-ΔLIC) measured − 0.39 (P = 0.01) for FCT formulation], while it was not significant for the other diagnoses. Comparison of iron overload variation between patients that received an FCT dose lower than the mean (range 6.4–15.3 mg/kg/day; N = 28) and those that received a higher one (range 15.6–28.2 mg/kg/day; N = 21) resulted in a significant difference in terms of ΔFTN (P = 0.001), but not ΔLIC (Fig. 3F). Gender, age, state of pubertal activation or BMI did not alter DFX PD significantly in this group of patients (Suppl. Tab. 5). Mean compliance measured 89.3 ± 12.1% and 91.6 ± 10.4% for DT and FCT, respectively, showing a limited but significant increase for the latter.

### Safety

**Figure 4:**
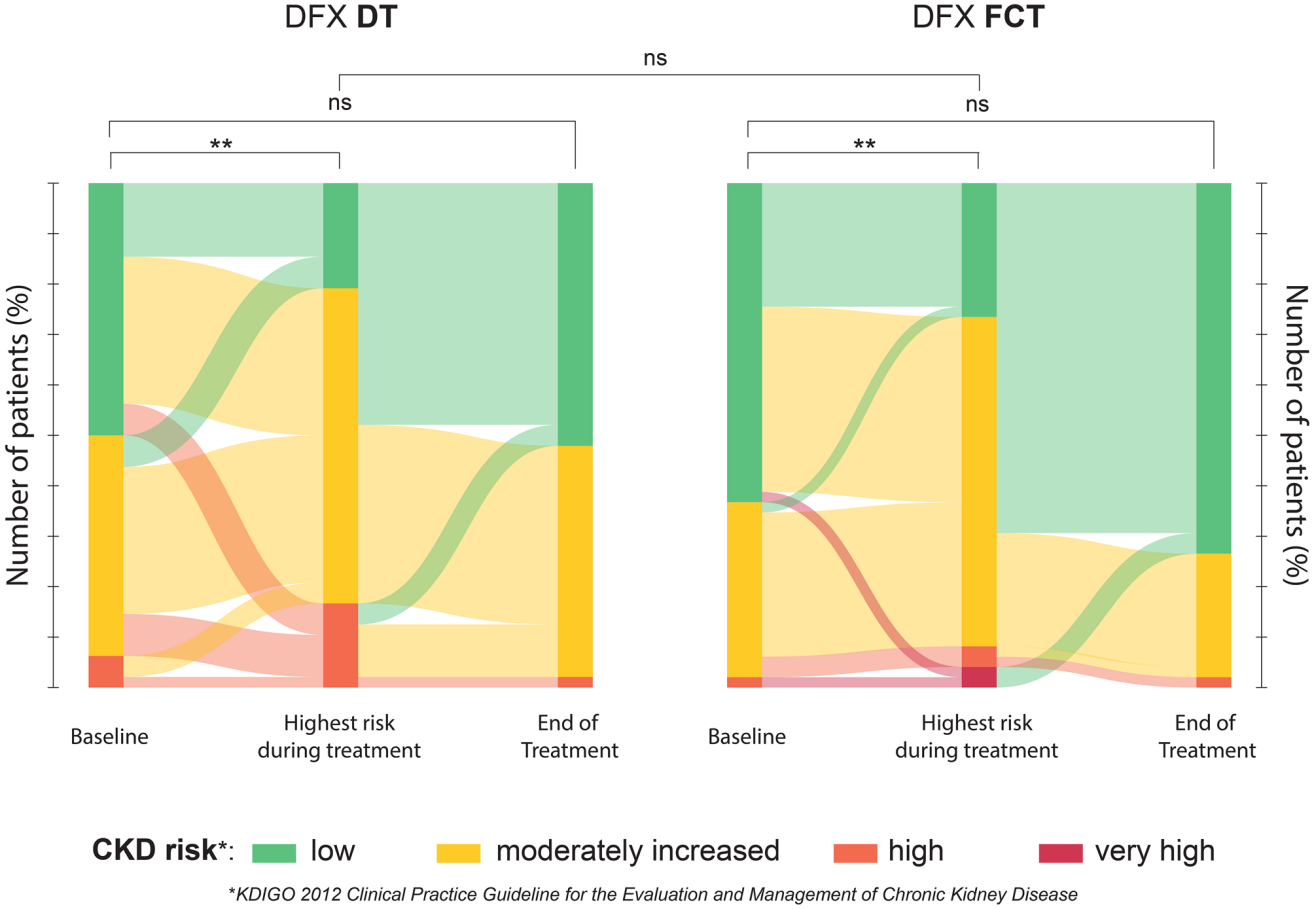
Chronic Kidney Disease (CKD) risk assessment. Sankey diagrams reproduce how patients were distributed among different CKD risk classes at baseline, at the highest risk reached during treatment and at the end of treatment considered in the study. For both DFX DT and FCT, the distribution within risk classes is not significantly different between the baseline and the end of treatment, but it is significantly shifted toward higher risk during treatment (P < 0.0001 (DT) and P = 0.0007 (FCT), one-sided test). The difference of probabilities of increasing CKD risk class during DFX treatment between DT and FCT formulation is not significant. *ns*: not significant.

Safety of DFX formulations was evaluated by the analysis of hepatic and renal function monitored during treatment. Baseline values were not different between formulations. Direct comparison of baseline to end-of-study values did not identify any relevant difference between formulations for both hepatic enzyme levels and sCr levels (Suppl. Fig. 5). No differences were observed considering each diagnosis separately. (Suppl. Tab. 6). Measuring AKI episodes based on eGFR reduction from baseline, we registered 1.06 ± 1.8 and 0.48 ± 1.9 episodes/patient/year during DT and FCT treatment, respectively (P = 0.02). Correlation of the number of AKI episodes with C_max_ or AUC was significant for FCT, but not DT (Suppl. Tab. 7). Furthermore, to evaluate the risk related to long-term DFX administration, CKD risk measured at baseline was compared with the highest CKD risk class reached during treatment. A significant increase was registered for both DT (P < 0.0001) and FCT treatment (P = 0.0007) and no differences were observed between the two formulations in terms of probability of CKD risk class worsening during treatment when taking FCT compared to DT. The mean time to reach the worst score during treatment was not different (12 ± 12 weeks for DT and 9 ± 11 weeks for FCT formulation). Relevantly, no significant differences were observed between baseline and end-of-treatment risk of CKD (Fig. 4).

## Discussion

DFX is the most recent iron chelator available to treat hemosiderosis; firstly released in DT formulation, it was later substituted by FCT formulation. However, only a few reports currently exist about long-term effects of DFX and, specifically, no independent data offer a direct and extensive comparison of the two formulations of DFX. We present here the first study analyzing pharmacokinetics (PK), pharmacodynamics (PD) and safety of subsequent formulations of DFX DT and DFX FCT in everyday clinical practice of iron overload treatment in β-thalassaemia and other rare anaemias.

Primarily, we found that DFX FCT was superior to DT in terms of both C_max_ and AUC, even at less than recommended dosage (− 41% compared to DT). Considering the indication of 30% lower dose of FCT compared to DT (or conversion factor 1.43) reported by the ECLIPSE study^21^, these results indicate an increased bioavailability and thus an exposure to the drug higher than expected for FCT when compared to DT formulation. We speculate that this difference could be likely due to different compositions between formulations. However, no additional data are available in this study to support this hypothesis and the absence of 24-h PK results from the ECLIPSE study limits a broader comparison. Interestingly, DFX FCT showed a higher correlation among dose and bioavailability parameters, namely C_max_ and AUC, indicating a better correspondence between administered dose and serum concentration. In addition, the variation coefficients of the mean were lower for FCT than DT for both C_max_ and AUC, in agreement with the results reported during the phase II study that led to FCT approval by FDA^21^. In terms of clinical practice, this supports the idea that less variability should be expected in the therapeutic effect observed when a selected dose is administered, allowing for more accurate regulation of the therapy, or scalability. Notably, since all the patients included in the study were subsequently treated with both formulations and matched, we speculate that the differences here reported are mostly due to inter-patient variability. In addition, the consistency of results among different diagnoses indicates a minor contribution of the underlying pathology in determining the response to the drug. For both formulations, measurements of DFX bioavailability at a given dose remained consistent regardless of gender or age, considered both as nominal age and as state of pubertal activation, potentially excluding the need of adjusting the dose according to these parameters. However, even if no differences were observed in the pharmacokinetic profile of the limited group of paediatric patients considered, recent reports^20,30,31^ highlighted the critical role of surveillance for this category.

Furthermore, the PD study provided data about the impact of DFX treatment under real-world conditions. To overcome a possible bias due to partially different conditions at baseline for the two study periods (namely, higher levels of iron overload measured by LIC, but not FTN, when starting FCT), the rate of variation instead of the total amount of iron overload was considered. As expected, no significant differences were observed when patients were treated with DFX DT or FCT, underlining an effective dynamic equilibrium among iron loading and elimination through chelation. The effect was confirmed to be dose-dependent for FCT formulation, but not DT, as demonstrated by the correlation between the administered dose and the efficacy parameters ΔFTN and ΔLIC. When considering subgroups of patients with the same diagnosis, this correlation was confirmed only for thalassaemia major, but the statistical analysis for the other groups was considerably limited by the small sample size. The moderate correlation coefficients measured even in the presence of a highly consistent pairing, however, indicate that the drug dose alone is not sufficient to predict its effect, suggesting a role of other unknown effect-modifiers. Further studies will be required to properly investigate and address the characteristics of these factors. The decrease in FTN observed in patients that took a FCT dose above the mean of all doses administered during the study (ΔFTN = − 504 ± 1216 ng/mL/year at mean dose of 20.2 mg/kg, N = 21) is consistent with the long-term results from patients that took part in the ECLIPSE study (ΔFTN = − 618 ± 1013 ng/ml in 12 months at mean dose of 21.3 mg/kg, N = 36)^32^. Altogether, these results indicate and confirm that DFX FCT is reliable in terms of dose adjusting and effects, thus suggesting the possibility of its better scalability compared to DT, as already observed in the PK analysis for this formulation.

Compliance to DFX DT was already close to optimal in the considered series and was slightly but consistently higher for FCT, confirming what was expected from previous reports about the acceptability of this formulation^21,23,32,33^. The evaluation of the impact of DFX FCT on compliance for patients with non-optimal adherence to therapy or a possible psychological role of novelty itself in long-term treatment is beyond the scope of this work.

Finally, the safety profile of DFX therapy was evaluated to assess the effects of long-term exposition to the drug. In agreement with previous results^21,34^, hepatic and renal functions at the end of study did not differ significantly from the levels registered at baseline. However, the analysis of renal function throughout the whole study time highlighted a consistent increased risk of CKD during treatment, comparable for both formulations. Retrospective assessment of CKD risk class in patients treated with DFX showed a consistent increase during treatment; nevertheless, the final distribution of patients in risk classes did not differ from the baseline. In addition, we report here that the number of AKI episodes retrospectively measured by laboratory findings was consistently lower for DFX FCT and correlated significantly with both DFX FCT C_max_ and AUC, but not with the overall dose administered. On the one side, this result suggests a possible role of this formulation in limiting acute renal complications through a more reliable dose-scaling, as previously discussed. On the other side, it indicates that the evaluation of DFX PK profile provides a more accurate instrument to limit drug-related toxicity than considering the administered dose alone. It is indeed essential to consider that careful management of these patients allowed the rapid adaptation of the ongoing therapy to any relevant adverse event, adjusting the dose or even suspending it if necessary, thus limiting any possible toxicity. Taken together, these observations indicate that (1) DFX is associated with consistent renal function impairment during long-term treatment and (2) these alterations could be fully reversible when strict monitoring is maintained.

Evidence of both observations was already available for DT formulation, although limited to a few patients with a long follow-up time (up to 108 weeks)^35^ and a reversible, mild increase in sCr has been reported since the core registration trials^19,34^. Different studies are available for FCT and none of them extensively evaluated its effects on renal function in a direct confrontation with DT at the indicated corresponding dose. The ECLIPSE study was limited to 24 weeks^21^ and its long-term extension considered FCT only, evaluating sCr, creatinine clearance, and urinary protein/creatinine ratio for up to 2 years in a larger group of patients with very different diagnoses^32^, reporting no major concerns about renal safety. A single-centre study analyzed the switch from DFX DT to FCT in 74 patients with thalassaemia and other transfusion-dependent conditions from all ages and reported a modest rise in mean sCr, with 6 patients (8%) having a sCr increase of > 30% from baseline, and 5 out 6 returning to the normal range by dose adjustment^22^. A separate study conducted in patients with low iron overload did not show an effect on decreased renal function, considered as ≥ 2 × upper limits of sCr levels or eGFR estimated by EPI^24^. However, the presence of several cases of proximal tubular toxicity in paediatric and adult patients taking DFX^30,31,36,37^ underlined the limitations of sCr alone as a sufficient and reliable marker of kidney injury, drawing attention to the necessity of more sensitive evaluations^30,31,36–41^. Therefore, this study provides for the first time a long-term direct comparison of both formulations, confirming the overall safety profile of the newest and highlighting the need for constant and careful drug monitoring to avoid side effects associated with DFX FCT chronic administration.

Among the limitations of this study, we highlight that inclusion criteria did not consider patients that terminated DFX before completing ≥ 12 months of treatment due to any cause, including poor tolerability or drug-related adverse events. Since these occurrences were not considered in the present study, caution has to be taken when applying the results presented to a broader population. Also, we did not include in the study the evaluation of the clinical condition of patients in the time frame between each period considered for DT and FCT study. For this reason, we are not able to motivate or explain the possible differences observed at FCT baseline when compared to DT. In addition, evaluation of PD did not account for sources of iron intake other than blood transfusions, e.g. dietary iron absorption. Finally, the patient population showed some variability in terms of diagnosis, basal conditions, and time of observation: while on the one hand this mirrors the heterogeneity typical of a real-world setting, on the other it could limit the consistency of results. To this regard, confirming the analysis carried out so far on a larger sample of patients could be particularly beneficial for those affected by iron-loading conditions different from thalassaemia major, that are under-represented within this population. Altogether, considering the data and results here reported alongside the increasing and somehow contrasting reports related to DFX, we think that a broader evaluation of DFX safety and effectiveness besides the clinical trials would be necessary, to improve the appropriateness of its use in different pathologies and clinical settings, optimizing the ratio among effectiveness and toxicity and moving a step forward in treatment tailoring and personalization.

## Supplementary Information

Supplementary Information.

## Data Availability

The datasets generated during the current study are available from the corresponding author on reasonable request.
