# Supplementary material for: Pharmacological and clinical evaluation of deferasirox formulations for treatment tailoring
Source: Sci Rep. 2021 Jun 15;11:12581. doi: 10.1038/s41598-021-91983-w (PMC8206201; doi:10.1038/s41598-021-91983-w)
Supplement: Supplementary file 1 — Supplementary Information. [file 41598_2021_91983_MOESM1_ESM.pdf]

SUPPLEMENTARY MATERIAL

TITLE: Pharmacological and clinical evaluation of deferasirox formulations for treatment tailoring

AUTHORS: Andrea Piolatto* (1), Paola Berchialla (1), Sarah Allegra (2), Silvia De Francia (2), Giovanni Battista Ferrero (1), Antonio Piga (1), Filomena Longo (1)

*corresponding author:

andrea.piolatto@unito.it
Reference Centre for Haemoglobinopathies, Department of Clinical and Biological Sciences, University of Torino, Torino
at AOU San Luigi Gonzaga, reg. Gonzole 10, 10043, Orbassano (To), Italy

AFFILIATIONS: (1) Department of Clinical and Biological Sciences, University of Torino, Torino; (2) Clinical Pharmacology, Department of Clinical and Biological Sciences, University of Torino, Torino

Supplementary Figure 1

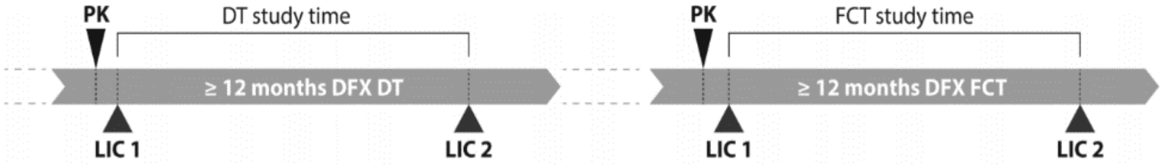

Suppl. Fig. 1. Study design. The scheme exemplifies the study design and timeline for pharmacokinetics (PK), pharmacodynamics (PD) and safety analysis. PK was performed before starting chelation therapy with DFX DT and then with DFX FCT. Liver iron concentration (LIC) was measured at the beginning (±1 month) of therapy with each formulation (LIC1) and then after approximately one year (LIC2), per clinical indication.

Supplementary Figure 2

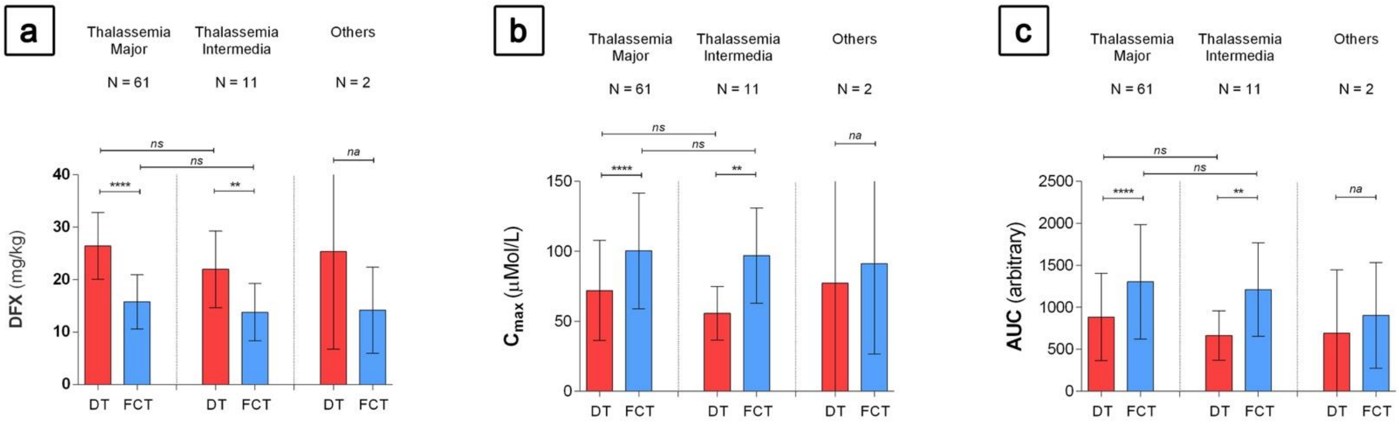

Suppl. Fig. 2. Pharmacokinetics (PK). Mean dose (**A**), maximum plasma concentration (Cmax) (**B**) and Area Under the Curve (AUC) (**C**) for PK curve at all doses, divided per diagnoses, for DFX DT and FCT. *na*: not available, too few data. Error bars: standard deviation.

Supplementary Figure 3

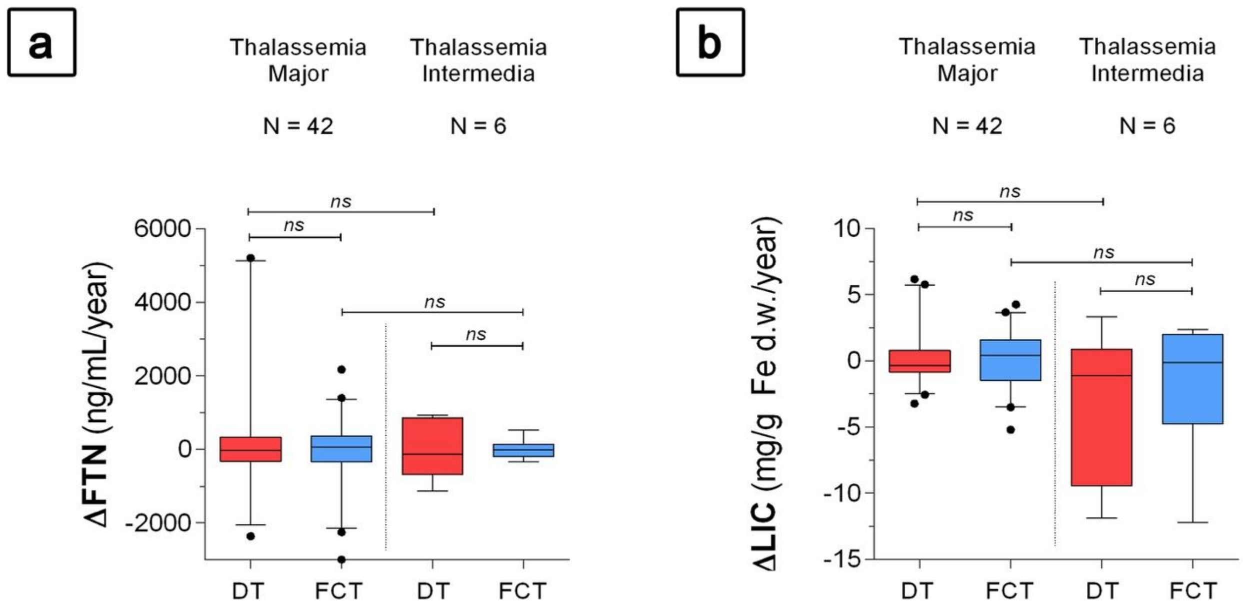

Suppl. Fig. 3. Pharmacodynamics (PD). Iron overload changing during treatment measured by yearly variation of serum ferritin (ΔFTN) (**a**) and LIC (ΔLIC) (**b**) levels, divided per diagnoses, for DFX DT and FCT. Whisker plots show median (line), 25 to 75 percentile (box), 5-95 percentile (error bar) and individual results out of previous ranges (dots). *ns*: not significant.

Supplementary Figure 4

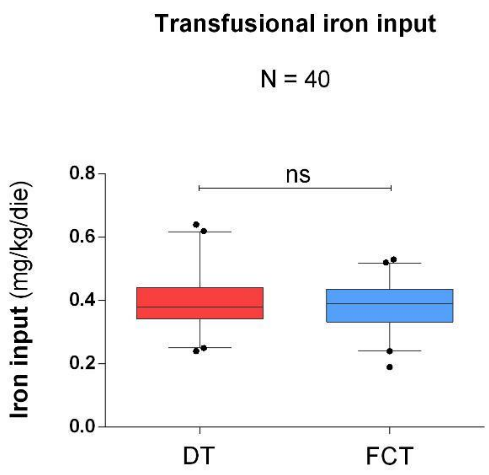

Suppl. Fig. 4. Transfusional iron input. Transfusional iron input was measured for transfusion-dependent patients in the PD and safety subgroup. No significant differences were observed. Whisker plots show median (line), 25 to 75 percentile (box), 5-95 percentile (error bar) and individual results out of previous ranges (dots).

Supplementary Figure 5

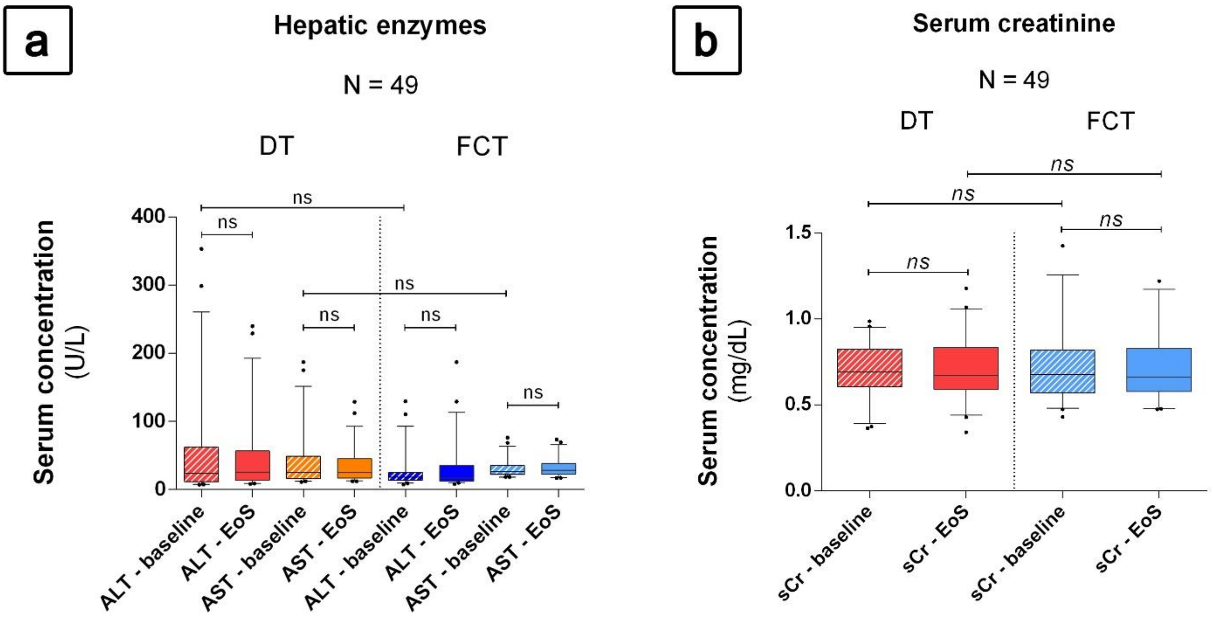

Suppl. Fig. 5. Safety. (**a**) Comparison of markers of hepatic toxicity alanine aminotransferase (ALT) and aspartate aminotransferase (AST), before starting treatment (baseline) and at the end of study (EoS) did not show any significant difference during each treatment period. (**b**) Similarly, measurement of serum creatinine (sCr) alone did not highlight any significant alteration. Whisker plots show median (line), 25 to 75 percentile (box), 5-95 percentile (error bar) and individual results out of previous ranges (dots).

Supplementary Table 1

		DT		FCT		
	N	*Mean*	*SD*	*Mean*	*SD*	*P*
Dose (mg/kg)						
Thalassemia Major	61	26.4	6.4	15.8	5.1	<0.0001
Thalassemia Intermedia	11	22.0	7.4	13.8	5.5	0.006
Others	2	25.4	18.6	14.2	8.2	*n/a*
C_{max} (µMol/L)						
Thalassemia Major	61	72	36	100	41	<0.0001
Thalassemia Intermedia	11	56	19	97	34	0.002
Others	2	77	87	91	65	*n/a*
AUC (arbitrary)						
Thalassemia Major	61	885	520	1302	680	<0.0001
Thalassemia Intermedia	11	661	295	1212	559	0.005
Others	2	690	757	903	631	*n/a*

Suppl. Tab. 1. Pharmacokinetics (PK). Deferasirox PK parameters are reported for each diagnosis of the patients considered in the study, i.e. Thalassemia Major, Thalassemia Intermedia and Others. No significant differences were observed among diagnoses. DT: dispersible tablets. FCT: film-coated tablets. *ns*: not significant. *n/a*: not available, too few data.

Supplementary Table 2

		DT		FCT	
	N	*Spearman's r*	*P*	*Spearman's r*	*P*
Dose- C_{max} correlation					
Thalassemia Major	61	0.24	*ns*	0.43	0.0005
Thalassemia Intermedia	11	0.21	*ns*	0.93	0.0001
Others	2	*n/a*	-	*n/a*	-
Dose- AUC correlation					
Thalassemia Major	61	0.20	*ns*	0.36	0.004
Thalassemia Intermedia	11	0.22	*ns*	0.76	0.01
Others	2	*n/a*	-	*n/a*	-

Suppl. Tab. 2. Pharmacokinetics (PK). Dose-C_{max} and dose-AUC correlation measured for each diagnosis. *ns*: not significant. *n/a*: not available, too few data.

Supplementary Table 3

	Cmax			AUC		
	coefficients	95%CI		coefficients	95%CI	
DT						
(Intercept)	-6.94144	-24.8815	42.72147	124.4912	-655.213	1063.078
DT dose mg/kg	2.3804	0.76342	3.02157	26.94455	-4.43369	42.64203
Age	0.29012	-1.10726	0.44987	-2.07112	-7.10109	9.00849
(Intercept)	6.05298	-44.6769	45.50508	-309.411	-816.271	805.8485
DT dose mg/kg	2.0358	0.36616	2.94094	31.80288	-3.61197	39.95402
BMI	0.04869	-1.01617	2.12649	11.63462	-10.301	49.36424
(Intercept)	5.61573	-17.2824	62.46972	-70.4167	-283.293	978.6087
DT dose mg/kg	2.10112	0.25961	3.05439	30.83333	-1.63723	39.70638
Sex (M vs F)	-0.84944	-18.9398	8.60331	40.33333	-375.839	318.0223
(Intercept)	-9.80714	-23.6812	57.21438	-210.276	-521.367	897.595
DT dose mg/kg	2.54762	0.78486	3.06821	35.32663	2.67022	40.38901
pubertal activation (Y vs N)	5.77857	-27.9512	24.33303	56.73367	-195.226	364.6466
FCT						
(Intercept)	13.02416	-7.43679	60.80738	-64.145	-379.234	441.5526
FCT dose mg/kg	4.05004	1.84606	6.01867	53.27637	7.3117	68.62964
Age	0.9307	0.33318	1.5997	14.76707	1.60467	21.57593
(Intercept)	-34.4349	-59.4291	-0.93954	-803.321	-1791.48	-358.347
FCT dose mg/kg	4.04779	2.84383	6.09771	53.58714	42.14472	73.58718
bmi	3.44154	1.67995	4.16992	58.5736	26.25247	88.57795
(Intercept)	51.94348	10.00625	75.2158	310.8652	49.66407	1063.118
FCT dose mg/kg	4.08696	3.25753	7.96843	69.1573	36.69086	81.83492
Sex (M vs F)	-26.5348	-48.8378	-5.44306	-215.691	-489.074	-47.9901
(Intercept)	38.41446	-21.1603	62.44909	364.6	-242.222	577.6957
FCT dose mg/kg	3.15663	1.42579	6.37969	54	26.83823	81.2669
pubertal activation (Y vs N)	12.57831	-6.73288	25.89879	97.4	20.80009	482.3605

Suppl. Tab. 3. Coefficients and 95% CI of quantile regression models between DT or FCT dose (mg/kg) and C_{max} and AUC adjusted respectively by age, BMI, sex and puberal activation. The analysis was performed using R software, version 4.0.2 (R Core Team (2020). R: A language and environment for statistical computing. R Fundation for Statistical Computing, Vienna, Austria).

Supplementary Table 4

		DT		FCT		
	N	*Mean*	*SD*	*Mean*	*SD*	*P*
ΔFTN (ng/mL/year)						
Thalassemia Major	42	234	1491	-72	1029	*ns*
Thalassemia Intermedia	6	47	839	12	288	*ns*
Others	1	*n/a*	-	*n/a*	-	*n/a*
ΔLIC (mg/g Fe dw/year)						
Thalassemia Major	42	0.2	2.1	0.1	2.0	*ns*
Thalassemia Intermedia	6	-3.2	5.8	-1.7	5.4	*ns*
Others	1	*n/a*	-	*n/a*	-	*n/a*

Suppl. Tab. 4. Pharmacodynamics (PD). Iron overload changing during treatment measured by yearly variation of serum ferritin (ΔFTN) and LIC (ΔLIC), for each diagnosis. *ns*: not significant. *n/a*: not available, too few data.

Supplementary Table 5

	ΔLIC			ΔFTN		
	coefficients	95%CI		coefficients	95%CI	
FCT						
(Intercept)	3.080	1.777	3.781	1146.608	269.300	1869.691
FCT dose mg/kg	-0.134	-0.208	-0.032	-52.799	-107.147	-25.930
Age	-0.012	-0.030	0.019	-8.036	-16.400	17.061
(Intercept)	4.232	0.589	6.125	1678.711	102.337	3020.841
FCT dose mg/kg	-0.134	-0.330	-0.092	-60.997	-115.572	-24.055
BMI	-0.068	-0.129	0.062	-29.266	-46.752	17.066
(Intercept)	3.115	1.108	3.718	873.397	304.517	1563.674
FCT dose mg/kg	-0.168	-0.196	-0.029	-61.239	-111.755	-27.353
Sex (M vs F)	-0.081	-0.880	0.810	197.569	30.732	408.437
(Intercept)	2.478	-10.524	4.147	1207.698	217.365	2009.802
FCT dose mg/kg	-0.142	-0.209	-0.028	-55.076	-101.112	-31.637
pubertal activation (Y vs N)	0.378	-0.643	13.059	-310.534	-1097.477	486.646

Suppl. Tab. 5. Coefficients and 95% CI of quantile regression models between DFX FCT dose (mg/kg) and ΔLIC (left) and ΔFTN (right) adjusted respectively by age at PK, BMI, sex and pubertal activation. Coefficients of DFX FCT dose represent the mean change in the median of ΔLIC and ΔFTN values after adjustment. Increase of one unit of DFX FCT dose is associated to a decrease of median ΔLIC values of-0.134 (95%CI-0.208;-0.032) and median ΔFTN values of-52.799 (95%CI:-107.147;-25.930). The analysis was performed using R software, version 4.0.2 (R Core Team (2020). R: A language and environment for statistical computing. R Fundation for Statistical Computing, Vienna, Austria).

Supplementary Table 6

		DT		FCT		
	N	*Mean*	*SD*	*Mean*	*SD*	*P*
Baseline ALT (U/l)						
Thalassemia Major	42	56	78	23	21	*ns*
Thalassemia Intermedia	6	32	26	34	14	*ns*
Others	1	*n/a*	-	*n/a*	-	*n/a*
Baseline AST (U/l)						
Thalassemia Major	42	41	42	30	12	*ns*
Thalassemia Intermedia	6	31	18	34	14	*ns*
Others	1	*n/a*	-	*n/a*	-	*n/a*
Baseline sCr (mg/dL)						
Thalassemia Major	42	0.7	0.2	0.9	1.5	*ns*
Thalassemia Intermedia	6	0.6	0.2	0.8	0.4	*ns*
Others	1	*n/a*	-	*n/a*	-	*n/a*
Baseline P/C ratio						
Thalassemia Major	42	0.19	0.10	0.18	0.12	*ns*
Thalassemia Intermedia	6	0.13	0.09	0.13	0.05	*ns*
Others	1	*n/a*	-	*n/a*	-	*n/a*

Suppl. Tab. 6. Safety. Baseline levels of safety parameters are reported for DT and FCT formulations. DT: dispersible tablets, FCT: film coated tablets, FTN: serum ferritin, LIC: liver iron concentration, ALT: alanine aminotransferase, AST: aspartate aminotransferase, sCr: serum creatine, P/C ratio: urinary protein to creatinine ratio. *ns*: not significant. *n/a*: not available, too few data.

Supplementary Table 7

		DT		FCT	
	N	*Spearman's r*	*P*	*Spearman's r*	*P*
AKI- C_{max} correlation	48	0.14	*ns*	0.47	0.0007
Dose- AUC correlation	48	0.20	*ns*	0.48	0.0006

Suppl. Tab. 7. Acute Kidney Injury (AKI). Correlation between the number of AKI retrospectively measured by lab eGFR and PK parameters, namely maximum plasma concentration (C_{max}) and Area Under the Curve (AUC). DT: dispersible tablets, FCT: film coated tablets. *ns*: not significant.
